# The effectiveness of web-based interventions on non-alcoholic fatty liver disease (NAFLD) in obese children: A study protocol for a randomized controlled trial

**DOI:** 10.3389/fpubh.2022.930901

**Published:** 2022-10-20

**Authors:** Caixia Tian, Jinliang Xu, Guofen Wang, Lidi Yu, Xiaoli Tang

**Affiliations:** Shaoxing Maternity and Child Health Care Center, Shaoxing, China

**Keywords:** NAFLD, obese children, education, protocol, web

## Abstract

**Aim:**

Non-alcoholic fatty liver disease (NAFLD) is currently the most prevalent liver disease in the world, increasing the risk of cirrhosis and hepatocellular carcinoma, and contributing to the development of type 2 diabetes, cardiovascular disease, and chronic kidney disease. This study aims to carry out a web-based continuum of a care intervention model to provide comprehensive care interventions for obese children with NAFLD, to improve the effectiveness of treatment of children with NAFLD.

**Design:**

A 1-year single-blinded randomized clinical trial in hospital in Zhejiang Province.

**Methods:**

Eighty subjects will implement the program in a randomized order. The interventions for the control group mainly consisted of the routine distribution of health education materials and health education by holding health-themed lectures, and the preliminary proposed interventions including establishing management teams, regularly delivering related health knowledge, daily uploading of health intervention records, regular supervision and mutual encouragement, home visiting and psychological guidance. The primary outcomes are serum biomarkers such as alanine aminotransferase (ALT) and gamma-glutamyl transferase (GGT), aspartate aminotransferase, and imaging (liver ultrasound and magnetic resonance imaging). Second outcomes are: BMI, waist-to-hip ratio and quality of life. In addition, socio-demographic characteristics such as age, gender and ethnicity will be recorded. Children aged 7–18 years old and diagnosed with NAFLD will be included, patients will be not eligible if they do not agree to participate or are participating in other health intervention programs. This study was registered on ClinicalTrials.gov (NCT05527938).

**Results:**

Over the past 30 years, NAFLD has been recognized as one of the most common liver diseases in adults and children. The current studies have focused on promoting lifestyle changes in children with NASH by providing some education and advice to children and their families to improve the histological features of NASH and lose weight. Because of the convenience and efficiency of the internet can provide some new strategies and ways for lifestyle interventions for children with NAFLD. In addition, we have designed a high-quality RCT based on the SPIRIT guidelines, which also provides strong evidence in this area.

## Introduction

With the development of economy and the improvement of material life, diet and living habits continue to change (excessive food intake, lack of exercise, etc.), over the past 30 years, the prevalence of overweightness and obesity among children and adolescents worldwide has increased at an alarming rate (1, 2). Nearly 1 out of every 6 children or adolescents in the U.S. has a body mass index (BMI) for age and sex above the 95th percentile and is considered obese (3).

Studies have found that the occurrence of fatty liver is directly related to the degree of obesity (4), and in recent years, non-alcoholic fatty liver disease (NAFLD) in children has surpassed hepatitis B as the most common liver disease in China (5). In children, the overall prevalence of NAFLD is 3–10%, rising to 40–70% in obese children (6). The prevalence of NAFLD in Asian and Chinese children is 6.3 and 3.4%, respectively (7). NAFLD is a clinicopathological syndrome of chronic hepatic steatosis involving more than 5% of hepatocytes in children and adolescents under the age of 18 years, excluding alcohol consumption and other definite causative factors leading to chronic fat deposition in the liver, and is a metabolic stress closely related to insulin resistance and genetic susceptibility The spectrum of disease includes non-alcoholic hepatic fat deposition. The disease spectrum includes non-alcoholic fatty liver (NAFL), non-alcoholic steatohepatitis (NASH) and its associated liver fibrosis and cirrhosis. Although fatty liver is a benign lesion and generally has little effect on children, it is often closely related to hyperlipidemia. It may develop into NASH and end-stage liver disease if not adequately diagnosed and treated, which may pose a potential threat to health and even life after adulthood, such as coronary heart disease, hypertension, and diabetes. It is a very important and urgent problem to reduce the incidence of childhood obesity and NAFLD.

Obesity is an independent risk factor for NAFLD, and the detection rate of fatty liver in obese children in China is 23.33% (5). Investigation shows that overnutrition and lack of moderate exercise are the main causes of obesity and fatty liver formation in children. Adjusting the diet and changing the lifestyle are the main interventions to control and prevent childhood obesity (8, 9), children have poor self-control and often fail to adhere to the planned measures, requiring the cooperation of parents, teachers, and doctors to supervise the implementation. Traditional interventions, in the process of implementation, are difficult to achieve the desired results due to time and frequency constraints, parental coddling, and lack of long-term effective and high-frequency participation and guidance from professionals, low compliance of the affected children.

Information technology has been increasingly used in the medical industry, from medical information records query to remote consultation and treatment, information technology has made a huge change in the traditional medical industry. The web-based continuity of care model based on new Internet technology can break traditional follow-up services' time and space limitations, expand service supply, improve service efficiency, and precisely match the diversified and multi-level health needs of nursing service recipients (10, 11). In this study, we followed the pace of the times. We tried for the first time the web-based continuity of care intervention model to provide comprehensive nursing interventions for obese children with NAFLD, always tracking their performance status, enabling them to grasp the knowledge of healthy weight loss, develop good lifestyle habits, and reduce their weight, thus reducing the incidence of NAFLD in children.

## Methods

### Reporting method

Following the Standard Protocol Items: Recommendations for Interventional Trails (SPIRIT 2013) checklist (12).

### Study design and setting

**Figure 1:**
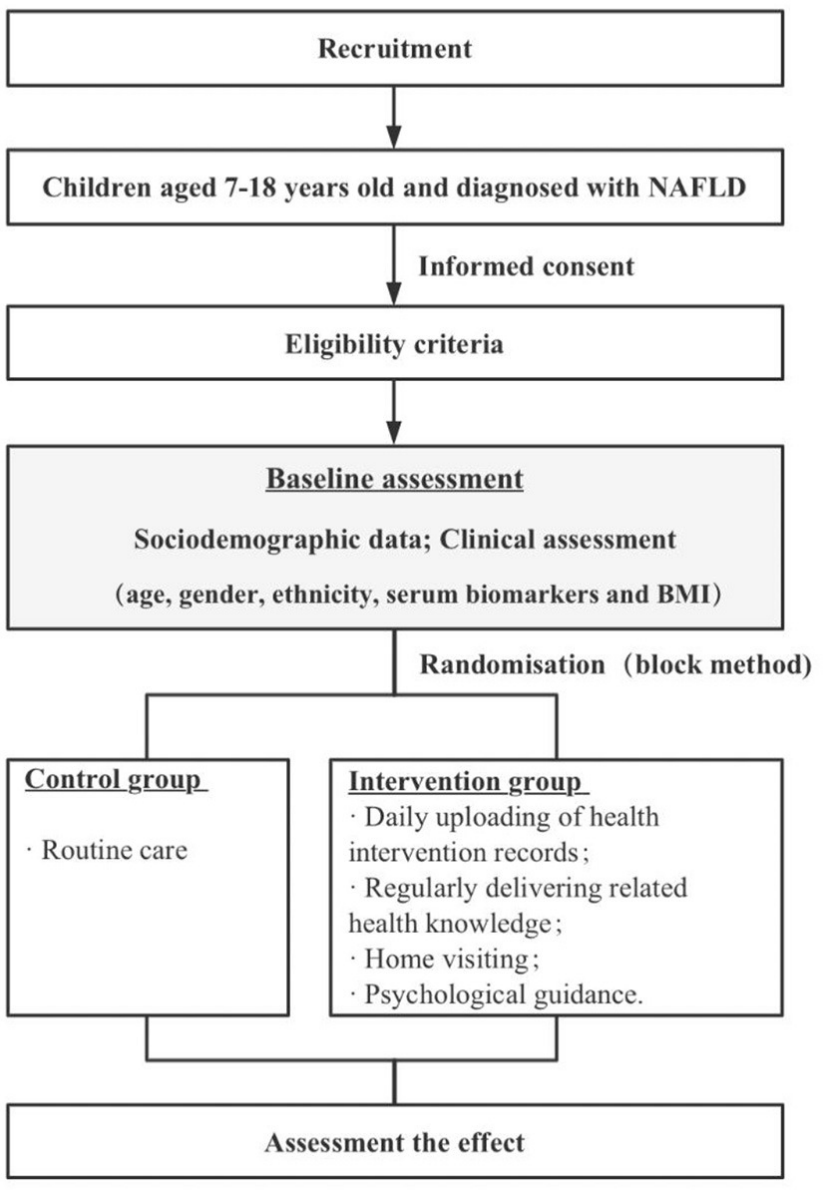
Schematic diagram of the study design.

We will conduct a 1-year single-blinded randomized clinical trial. To recruit more study subjects, the study will conduct at a large hospital in Zhejiang Province (Figure 1).

### Eligibility criteria for patients

In order to be eligible to participate in this study, patients should be: Children aged 7–18 years old and diagnosed with NAFLD: Referring to the Expert Consensus on the Diagnosis and Treatment of Non-Alcoholic Fatty Liver Disease in Children (13), the clinical diagnostic criteria need to meet 1–4 of the following, and any 1 of (5) or (6): (1) except for other pathological obesity; (2) in addition to the clinical manifestations of the primary disease, some patients may have non-specific symptoms and signs such as weakness, dyspepsia, vague pain in the liver area, hepatosplenomegaly, etc.; (3) overweight, obesity (centripetal obesity), elevated fasting glucose, lipid metabolism disorders, hypertension, and other metabolic syndromes; (4) alanine aminotransferase (ALT) is elevated more than 1.5 times the upper limit of normal value (60 U/L) and persists for more than 3 months; (5) liver imaging technique to diagnose hepatic steatosis; (6) histological changes in liver biopsy meet the pathological diagnostic criteria of fatty liver disease. Patients will not be eligible if they do not agree to participate or participate in other health intervention programs.

### Intervention

The interventions for the control group mainly consist of the routine distribution of health education materials and health education by holding health-themed lectures, and the preliminary proposed interventions are shown in Table 1.

**Table 1:** Control group.

**Intervention**	**Intervention content and frequency**
Routine care	At each visit to the hospital, in this time, the child and his parents are given health education on diet and exercise booklets, and the parents supervise the child's daily life. The team members will review the child's condition every month for feedback.

To the above, the following four intervention components will be added, which shown in Table 2.

**Table 2:** Intervention group.

**Intervention**	**Intervention content and frequency**
1. Establishing management teams	In addition to the subject leader, seven research team members will be each responsible for several participants (children with NAFLD) in need of intervention and formed a WeChat group for their parents.
2. Establishment of a nursing intervention team.	Composed of the Director of Nursing, a pediatric endocrinologist, a senior health lecturer and a nutritionist, three nurse leaders and a charge nurse with a master's degree. The director of the nursing department is the team leader. All members received half-month training from the team leader, including data collection, the purpose, model and implementation methods of the extended care intervention, and communication skills with the children and their parents. The training will be mainly in the form of intensive classes, and a theoretical examination will be conducted at the end of the training, requiring each member to pass.
3. Daily uploading of health intervention records	The nutritionist on the team develops personalized diet menu for each child based on age, height, weight, previous medical history, and any allergies, and at the same time, and an exercise therapist develops the tailored exercise plan for each NAFLD children (Appendix). The children and their parents upload the amount of exercise, and diet in the WeChat group every day, all parents can monitor and encourage each other in the group. Questionnaires are regularly posted in the group to judge the intervention effect and to adjust the implementation plan according to the individual intervention effect, with the aim of optimizing the plan and thus personalizing the intervention, and for subsequent studies we will consider using apps or WeChat applets for the intervention.
4. Regularly delivering related health knowledge.	Management team members Forming a WeChat group of children with NAFLD family members. Regularly through the WeChat platform, research team send relevant promotional materials such as nutrition knowledge and exercise knowledge to the children and parents 5 times per week, and we can also distribute some PPTs and videos of the professional nursing staff's promotional knowledge.
5. Home visiting	Members of the group made regular home visits, usually once every 2 weeks. The purpose of the visit is to grasp the development of the child's condition and to provide health education and dietary guidance to the child based on the visit results. If there is no one at home, it will be accessed by phone.
6. Psychological guidance.	The health teachers in the team should closely understand the psychological trends of the patients during the telephone follow-up and home visits and verbally communicate more with the children to stimulate them to develop good living habits and increase their confidence in healing.

### Outcome

The primary outcome are serum biomarkers such as alanine aminotransferase (ALT) and gamma-glutamyl transferase (GGT). Second outcomes are: aspartate aminotransferase, and liver imaging (liver ultrasound and magnetic resonance imaging), BMI, waist-to-hip ratio and quality of life. In addition, socio-demographic characteristics such as age, gender, and ethnicity will be recorded. All outcomes were measured at baseline, week 4, week 16, week 24, week 36, and week 48 to determine the trajectory of change in outcome variables over intervention process.

### Participant timeline

Baseline characteristics of the patient (e.g., age, gender, ethnicity, serum biomarkers, and BMI) will be collected.

### Sample size

Sample size estimation will be performed using Professional Association for SQL Server (PASS) 2014 software for grasp degree analysis (Power Analysis). The mean effect size of the lifestyle intervention on the improvement of ALT indicators (Effect size) was estimated to be −1.35 according to the Meta-analysis of the clinical study conducted by Utz-Melere et al. (14, 15). The sample size required will be 32 study subjects per group by power analysis in reaching 80% effectiveness, using a two-tailed test with a test level of 0.05. Based on a 20% failure rate, this part of the study required 80 subjects, 40 in each group.

### Recruitment

In this study, participants will be recruited through two pathways. First, through the assistance of nurse managers of relevant departments in hospitals has been established to recruit, post or place leaflets, posters in departments of hospitals, and have researchers promote the project after the daily treatment of patients to attract interested patients. Secondly, through network: Release and diffuse recruitment information through WeChat friend circle, WeChat group, Weibo and other network platforms.

### Assignment of intervention

Before randomization grouping, the children's medical records will be read carefully to obtain information about their demographics and diseases. Patients will be numbered according to the enrollment order and assigned to the experimental and control groups according to the random number table method. Statistical analyses will be performed to compare the balance of the two groups in terms of age, sex, ALT, BMI, and quality of life scores. The child's parents are entered into the same group as the child.

### Blinding

Study personnel involved in the data assessment of the study are blinded to the patients' treatment assignment. The clinical doctors that deliver the study treatment are not blinded and are therefore uninvolved in the patients' data assessments related to this study. Data analysts are blinded to group allocation.

### Data collection and management

First, members of the research team explained the purpose and significance of the study to the participants and obtained consent and support. Then, subjects who met the inclusion criteria were selected and the purpose, content, and significance of the survey were explained to them, explaining that the study results would be used only for scientific research and that medical confidentiality would be strictly observed. After that, the investigator personally distributed the questionnaire, and the patients filled it out under a uniform instructional language. For patients with no reading ability or writing difficulty but could understand and answer the questions correctly, the researcher will repeat the questions and options in a neutral tone, and the patients themselves chose. The researcher assisted in filling out the questionnaire, which will be distributed and collected on the spot. The questionnaires took about 25–30 min to fill out; finally, the researcher carefully check the collected questionnaires and promptly eliminated invalid ones. In addition, anthropometric measurements [length, weight, waist circumference (waist circumference) and blood pressure], blood sampling [serum alanine aminotransferase (ALT), insulin, glucose and lipids] and 1H-MRS were taken to determine hepatic steatosis. Use the collected height and weight data to calculate age-adjusted BMI-z scores (16).

### Statistical analyses

First, after eliminating the questionnaires that did not meet the requirements, all data will be organized and quantified and then entered the statistical software in pairs for analysis. Then statistical description of the data will be done, including descriptive statistical analysis of patients' general information, the mean and standard deviation will be used to describe the measurement data that conformed to normal distribution. Finally, statistical inferences will be made and *t*-test, ANOVA. The effect of the intervention on NAFLD w analyzed using McNemar's test for paired categorical data and χ^2^ or Fisher's exact test for independent categorical data, as appropriate. Changes in continuous variables will be analyzed using paired Student *t*-test. Statistical significance will be set at *P* < 0.05. Using analysis of covariance we evaluated whether baseline variables predicted changes in liver fat content and ALT during 6 months of treatment.

### Ethics approval and consent to participate

This study protocol will be conducted at the study site only after obtaining review permission from the hospital administration where the study participants will be enrolled. The investigator must obtain informed consent from the participant and minimize the participant's exposure to other risks. Before the start of the study, the investigator introduced himself or herself and fully informed participants of the content and purpose of the study so that they were aware of the behaviors they might be asked to cooperate with and the amount of time they would spend in the study. The investigator informed participants that the study would not provide them with any realistic benefits, but also no disadvantages or risks; participants were informed that all recorded material would be used for research purposes only. Participants participated in the study anonymously in a WeChat group, where each person was required to change their name (replaced by a serial number) after joining the group. Participation was voluntary and participants could opt out of this study at any time. Verbal informed consent will be obtained from the participant before the start of the formal interview. The real names of the participants will be not used in the data and study reports; instead, numbers will be used to identify the participants. This study was registered on ClinicalTrials.gov (NCT05527938).

## Discussion

NAFLD is currently the most prevalent liver disease in the world, increasing the risk of cirrhosis and hepatocellular carcinoma, and contributing to the development of type 2 diabetes, cardiovascular disease, and chronic kidney disease (17, 18). BMI remains an important risk factor for NAFLD in children and young adults, and adipose tissue dysfunction contributes to the pathogenesis of NAFLD (19). General healthy eating advice and physical activity are recommended to promote weight loss in pediatric NAFLD. However, lifestyle interventions, be it general caloric restriction diets or low-fat or low-carbohydrate diets with or without increased physical activity, are frequently afflicted with low compliance and high drop-out and relapse rates in children and adults (20–22). In this study, we will implement the web-based intervention to provide more social and peer support to the affected child and family to improve their compliance with the intervention.

This current study has several strong features. Firstly, life-style interventions are the first step in managing children with NAFLD (14). The current studies have focused on promoting lifestyle changes in children with NASH by providing some education and advice to children and their families to improve the histological features of NASH and lose weight. As a previous systematic review mentioned, there is significant heterogeneity in study design quality, sample size, duration, outcome measures, and treatment interventions in RCTs for children with NAFLD. There is also a lack of effective and well-established lifestyle intervention programs to explore improvement in NAFLD. In addition, to our knowledge, web-based studies are currently being applied in adult NAFLD, providing web-based online training for those unable to attend face-to-face or group education (23). However, compared to adults, the child population is less sensitive to the Internet and adherent. Then, interventions for the entire population of children with NAFLD were not appropriately graded according to the severity of the disease, much less individualized for the different levels of intervention. Therefore, we used an adaptive strategy through Internet technology to implement individualized interventions according to each child's disease severity.

There are also some limitations that need to be addressed. Firstly, we estimate that there will be an attrition rate of ~10% of children who enroll in the study, mostly due to financial issues or time constraints. To minimize the effect of the biases, we will conduct the analysis on an intention-to-treat basis. It is important to consider that although this study will be conducted in the hepatobiliary ward of a specialist women's and children's hospital in Shaoxing, Zhejiang Province, this may lead to limitations in the findings. However, we are conducting a formative evaluation throughout the implementation of the project to explore which interventions or environmental factors may hinder or contribute to the effectiveness of this project at different stages of implementation to facilitate global replication.

## Author contributions

CT, XT, and GW: systematic concept and designed. CT, XT, and LY: analysis the data. CT and XT: drifting the manuscript. All authors: revised the manuscript. All authors have read and approved the submission of this manuscript.

## Funding

This work was supported by the Science Technology Department of Shaoxing, China (Grant No. 2020A13033).

## Conflict of interest

The authors declare that the research was conducted in the absence of any commercial or financial relationships that could be construed as a potential conflict of interest.

## Publisher's note

All claims expressed in this article are solely those of the authors and do not necessarily represent those of their affiliated organizations, or those of the publisher, the editors and the reviewers. Any product that may be evaluated in this article, or claim that may be made by its manufacturer, is not guaranteed or endorsed by the publisher.
