# Supplementary material for: The effectiveness of web-based interventions on non-alcoholic fatty liver disease (NAFLD) in obese children: A study protocol for a randomized controlled trial
Source: Front Public Health. 2022 Oct 20;10:930901. doi: 10.3389/fpubh.2022.930901 (PMC9632618; doi:10.3389/fpubh.2022.930901)
Supplement: Supplementary file 1 [file Data_Sheet_1.PDF]

Appendix: Personalized diet plan

XX: 11 weeks old, body mass index 27.81, moderately obese, fatty liver, insulin resistance, breath, little water, little exercise, adjust daily energy 1650kcal during the weight loss period, do not eat egg yolk for the time being, use soy products instead, preferably use olive oil or camellia oil, can bring biscuits to school, so only add milk in the evening, pay special attention to drink more water, exercise choice of cycling, hula hoop, walking, etc., do not add sugar when cooking vegetables, do not add cantaloupe, lychee, mango, durian, melon and other fruits with high sugar content. Don't add sugar to dishes, and don't eat fruits with high sugar content such as cantaloupe, cinnamon, lychee, mango, durian, and watermelon.

Table 1 Daily food intake

	All day (g)	Breakfast	Morning Extra Meal	Lunch	Dinner	Extra meal (before 8 p.m. or when picking up children after school)
Cereals	150	50	25	75	50	0
Meat	100	0	0	50	50	0
Eggs	25(yolk removed)	25	0	0	0	0
Milk	300	200(low-fat milk)	0	0	0	100(Yoghurt)
Soybeans	50	25	0	0	25	0
Vegetables	500	200	0	100	200	0
Fruits	250	100	0	0	0	150g(before 8pm)
Edible oils	25	5	0	10	10	0

Note: The above ingredients are all raw weight, as we don't know the Chinese food situation at school, please teach your children that 75g of rice is equivalent to a flat bowl with a diameter of 13cm, 100g of meat is equivalent to the size of your mother's palm, if the school stir-fry is oily, it is recommended to eat with warm boiled water, if you can't eat enough, you can add a moderate amount of vegetables to the three meals, in order to achieve the weight loss effect, drink 2000ml of water daily, it is best to insist on 5000 steps after dinner, or do other exercises, no food after 20:00, if you are really hungry add meals a little later, it is recommended that fruit is best eaten in the evening when you pick up, drink milk at night, drink water in moderation when you are hungry.

Table 2 Weekly diet menu

Date	Breakfast	Morning Extra Meal	Afternoon Extra Meal	Dinner	Extra meal (before 8 p.m. or when picking up children after school)
Monday	Yam and millet porridge (small amount of yam, 25g of millet), meat bun (40g); boiled egg (25g of egg white), fried string beans and mushrooms (70g of string beans, 30g of mushrooms), low-fat milk 200ml, 100g of small tomatoes, 2 slices of boiled dried tofu.	Soda crackers 25g		Purple rice (purple rice 50g), braised tofu (southern tofu 150g), fried shredded pork with green pepper (shredded pork 50g, green pepper 100g), fried shrimp skin with cabbage (cabbage 100g, shrimp skin a little), winter melon and barley soup	Tangerine 150g, yoghurt 100g.
Tuesday	Wholemeal bread 30g, white porridge (rice 25g), low-fat milk 200ml, shredded courgetti stir-fried with dried tofu (dried tofu 25g, courgetti 100g) boiled egg (egg white 25g), cucumber 100g.	Biscuits 25g		125g steamed yam, rice (25g japonica rice), shredded beef and dried tofu stir-fried with asparagus (25g shredded beef, 25g dried tofu, 100g asparagus), shredded seaweed in cold sauce (100g seaweed), 3 prawns in brine (40g). Tomato soup with winter melon	Yoghurt apple kiwi platter (100g yoghurt, 150g fruit).
Wednesday	Black rice porridge (25g black rice), 40g steamed buns, 200ml soy milk, fried wild rice with green pepper and onion (50g green pepper, 25g onion and 25g wild rice). Marinated egg in shell (25g egg white), 150g apple.	Whole meal bread 30g		6-7 dumplings, cold black fungus (100g water-haired fungus), shredded tofu with celery and enoki mushrooms (80g celery, 20g enoki mushrooms, 35g shredded tofu)<br>Silky gourd and duck blood tofu soup	Cucumber 100g, low fat milk 300ml.
Thursday	Steamed potatoes 100g, small meat buns 40g, small low fat milk 200ml, marinated quail eggs (four with yolks removed), stir-fried mushrooms with shredded cabbage and dried tofu (75g cabbage, 25g shredded tofu, 25g mushrooms), small tomatoes 100g.	Soda Crackers 25g		175g corn, rice (25g japonica rice), fried tofu (150g young tofu), amaranth and enoki mushrooms (150g amaranth, 50g enoki mushrooms), steamed crucian carp (80g crucian carp), puzi tomato soup.	Fruit salad with yoghurt (100g yoghurt, 150g fruit).
Friday	White porridge (25g japonica rice), 125g steamed yam, 100g yoghurt, scrambled	Whole meal bread 30g		Oatmeal rice (20g oats, 30g japonica rice), chicken breast with fried carrot and dried	Apple 100g, low fat milk 200ml.

	eggs with tomatoes (100g tomatoes, 50g eggs), 2 slices of marinated dried tofu.			tofu and asparagus (50g chicken breast, 10g carrot, 50g asparagus, 25g dried tofu), braised winter melon, (50g winter melon), Bean sprout and shrimp skin with seaweed soup.	
Saturday	Rice (50g japonica rice), boiled egg (25g egg white, 200ml low fat milk, shredded cabbage and dried tofu (200g cabbage, barley and red bean soup (5g barley, 25g red beans), 100g sainted fruit.	Fruit salad with yoghurt(100g yoghurt, 150g fruit).	Soda Crackers 25g	Steamed sweet potato 100g, rice (japonica rice 25g), shredded lettuce, mushroom and dried tofu (lettuce 50g, mushroom 50g, dried tofu 25g), fried shrimp with loofah (loofah 100g, shrimp 50g).Broccoli, shredded pork and yam soup (shredded pork, small amount of yam).	
Sunday	Millet and red dates porridge (50g japonica rice), 30g wholemeal bread, fried tofu with greens (100g greens, 75g old tofu), boiled egg (25g egg white), 200ml low fat milk, 100g loquat.	Soda Crackers 25g	Fruit salad with yoghurt(100g yoghurt, 150g fruit).	Steamed corn 175g, rice (japonica rice 25g), marinated beef (beef 50g), stir-fried lettuce (lettuce 100g), stir-fried wild rice with long cowpeas (long cowpeas 80g, wild rice 20g) winter melon and tomato squash soup.	

Note: The child will be served a standard school meal at school, so lunch menus are not available
